# Designing an Educational and Training Program for Diabetes Health Educators at Diabetic Health Centers, Khartoum State, Sudan; 2007-2010

**DOI:** 10.5539/gjhs.v5n5p207

**Published:** 2013-07-23

**Authors:** Fathia Osman MakkiAwouda, Taha Ahmed Elmukashfi, Seed Ahmed Hag Al-Tom

**Affiliations:** 1 Faculty of Nursing Sciences, University of Khartoum, Khartoum, Sudan; 2 Department of Community Medicine, Faculty of Medicine, University of Khartoum, Khartoum, Sudan; 3 Curriculum Department, Faculty of Education, University of Khartoum, Khartoum, Sudan

**Keywords:** diabetes health educators, diabetic health centers, Khartoum State, Sudan

## Abstract

**Background:**

By the year 2030 the number of diabetic patients is expected to reach 366 million worldwide (World Health Organization [WHO], 2013).

**Methods:**

It was an intervention-facility based. The study focused on designing and implementing an educational and training program for health educators and to assess its effects on achievements of diabetes health educators; at Diabetic Health Centers in Khartoum State, Sudan; 2007-2010. The study population composed of diabetes health educators working in Diabetic Health Centers. Their total number was (36) and all were included in the study. Pre and post tests were done. Data was entered using SPSS. Parametric methods including t-test to assess result of training program before and after intervention were used. P-value ≤ 0.05 was considered statistically significant.

**Results:**

Thirty six diabetes health educators attended the program. There was a significant statistical difference (p=0.001) between mean of Pre and post test concerning knowledge of diabetes health educators. To my knowledge there were no studies done, before, in Sudan to evaluate knowledge of diabetes health educators after attending an educational program in order to compare results with.

**Conclusion and Recommendations:**

The designed education program improved health educators’ knowledge. Educating diabetes health educators is important in program of diabetes management. In-serving training of diabetes health educators, aiding diabetic health centers with educational materials, and more research on evaluation of impact of education on diabetes health educators; was recommended.

## 1. Introduction

Diabetes mellitus is a chronic multisystem disease related to abnormal insulin production, impaired insulin utilization, or both. Diabetes mellitus is a serious health problem throughout the world and its prevalence is increasing rapidly (Lewis, Heitkemper, Dirksen, O’ Brien & Bucher, 2007).

In the United States an estimated number of 25.8 million Americans were affected which represents 8.3% of the total population, and about 1.9 million new cases of diabetes were diagnosed among people aged 20 years and over. The total number of deaths due to diabetes in USA was 231,404 (American Diabetes Association [ADA], 2011).

In Africa, by the year of 2030 the number of diabetic patients in Nigeria expected to be 4.835000, followed by South Africa (1,286,000) and Ethiopia (1,820,000). The total diabetic patients in all African countries expected to be 18,234,000. Regarding Eastern Mediterranean region; taking Pakistan as an example, by the year 2030 the number of diabetic patients will be 13,853,000; while the total number in the region expected to be 42,600,000. In Sudan the number of diabetic patients during year 2000 was 447,000, but in the year 2030 the number expected to be 1,277,000 (WHO, 2013).

Whatever drug is taken (oral pills or insulin) patient should know the name and dose of medication, when to take it, and side effects that can occur and how to manage by himself (Edelmen & Gattillo, 2009)

*Two types of diabetes were observed*. Type 1 diabetes: occurs before the age of 40. People with this type treated with insulin injections. Type 2 diabetes: occurs after the age of 40. The pancreas still produces insulin, but it is amount is not enough and at the same time the body resists the produced insulin. So, there is high blood glucose level (Lewis et al., 2007).

Education plays a big role in management of the disease and its complications. Education is not only giving information, but when given properly liberates the human being (Donohue-Porter 2009).

As stated by Smeltzer & Bare (2004) the teaching plan has to organize information and skills into two main types:

i Basic or survival skills and information
ii In depth (advanced) or continuing education

The design of any educational program needs to determine the goals, the teaching –learning objectives, content, teaching methods or strategy and evaluation. The diabetes educational program should include all the above components. (Basavathappa, 2009)

*Training:* It is the systematic modification of behavior through learning planned experience. As stated by Jerry& Steven 2006 there are seven principles to carry out an effective training program, and these are: -

1). The principle of the teaching specialist. A teaching specialist must know the program, lesson, subject, skill, or truth to be taught
2). The principle of the learner. A learner must attend with interest to the program, lesson, or subject.
3). The principle of the language. The language used as a medium between the teaching specialist and the learner must be common to both.
4). The principle of the lesson. The information or skill to be mastered must be explicable in terms of information already known by the learner — the unknown must be explained by means of the known.
5). The principle of the teaching process. The teaching process must be arousing, using the learner's mind to grasp the desired thought or to master the desired skill.
6). The principle of the learning process. The learning process must turn one's own understanding of a new idea or truth into an overt habit that demonstrates the new awareness.
7). The principle of review and application. The evidence of individual development must be reflected through a reviewing, rethinking, reproduction, and applying of the material, information, truth, or skill that has been communicated.

### 1.1 Problem Statement

During the period 2007 – 2009, a manual for educating diabetes health educators in Diabetic Health Centers in Khartoum State, Sudan, in order to raise their knowledge and awareness regarding diabetic management; was designed and implemented.

### 1.2 General Objective

To design and implement an educational program for diabetes health educators who were working in Diabetic Health Centers in Khartoum State; and to study the effect of the designed program on their achievement; 2007 - 2009.

### 1.3 Specific Objectives

1). To determine the level of knowledge of diabetes health educators working in Diabetic Health Centers in Khartoum State; Sudan; before and after implementation of the program regarding the different aspects of diabetic health care.
2). To design educational and training program for diabetes health educators who were working in Diabetic Centers in Khartoum State; Sudan; based on the pre test for them and the literature of training educators.
3). To determine the effect of the educational program in the achievement of diabetes health educators’ knowledge; Diabetic Centers in Khartoum State; Sudan.

## 2. Methodology

### 2.1 Study Design

It was an interventional facility based study.

### 2.2 Study Area

Khartoum State is one of the 17 states of Sudan. It is the political and commercial center of Sudan. It has an area of 22736 Km² and almost situated in the center of Sudan. It lies between latitudes 8.45 degrees & 23.8 degrees north and longitudes 21.49 degrees to 38.24 degrees east. The most prominent feature is the Blue and White Nile which form the main stream of the River Nile. The state has been divided into seven localities. The areas of intervention of the study consist of four health centers: (1) Jabir Abu Aliz: is the first specialized governmental diabetes health center in Sudan. It was established in August 1998 in Khartoum State. The center receives more than 250 patients daily. The registered number up to November 2009 is 35,000. The goals of Jabir Abu Aliz Diabetics’ Clinic are: Screening high-risk group; educating the patients as well as their relatives about all aspects of D.M. mainly diabetic neuropathy, retinopathy and vascular diseases; insuring availability of medicines as far as possible; training for all health professionals from different states of Sudan. (2) Soba University Hospital: It was established in the year 1975 as the first university hospital in Sudan. The objective of this hospital is training and qualifying all medical and health professionals. The hospital includes most of the therapeutic departments for medical specialties. The diabetic clinic in this hospital works as an outpatient clinic three times per week. (3) Khartoum North Teaching Hospital: It was established in 1960 in Khartoum North. The hospital receives all critical and emergency cases in the emergency department for the first 24 hours. The hospital includes therapeutic departments for most medical specialties, which form training centers for doctors and other medical professionals and nursing. The diabetic clinic does not start yet. (4) Almerum Omdurman clinic: It was started in 2008 and was closed in less than one year.

### 2.3 Study Population

The study population was composed of Diabetes Health Educators: They are health care professionals who have mastered knowledge and skills in the biological and social sciences, communication, counseling, education and have experience in care of people with diabetes. They are nurses graduated from colleges of nursing with BSc. of nursing sciences, working in diabetic health centers; in Khartoum State; Sudan. Their total number was 36 attended the pre and post tests. Inclusion criteria of Diabetes Health Educators: (i) Graduate from colleges of nursing sciences. (ii) Working in Diabetic Health Centers in Khartoum State; Sudan. Exclusion criteria of Diabetes Health Educators: (i) Non-graduate from college of nursing science. (ii) Not working in Diabetic Health Centers in Khartoum State, Sudan.

### 2.4 Sample Size

Total coverage of 36 nurses working in Diabetic Health Centers in Khartoum State, Sudan.

### 2.5 Tools of the Study

The tools of the study were as follows: - (i) Pre and post tests for diabetes health educators. (ii) The educational program (intervention).

### 2.6 The Educational Program (Intervention)

The designed program is based on the pre test of diabetes health educators and the literature of educating and training of educators, it consists of the following: -

### 2.7 The Objectives of the Program

Cover the main domains, which are cognitive, skills, psychomotor and attitudes.

### 2.8 Contents of the Program

The components of the educational program are:

1. Introductory sessions about objectives, teaching methods and evaluation.
2. Simple patho-physiology: a. Basic definition of diabetes mellitus. b. Normal blood glucose ranges and target blood glucose levels. c. Effects of insulin and exercise. d. Effects of food, stress including illness and infections. e. Basic treatment approaches
3. Treatment modalities: a. Administration of insulin and oral anti –diabetes medications. b. Diet information. c. Monitoring blood glucose and ketones.
4. Recognitions, treatment and prevention of acute complications: a. Hypoglycemia. b. Hyperglycemia.

The program composed of 4 units in 10 sessions, covering major aspects of diabetes mellitus and was approved by the technical and ethical committee.

### 2.9 Teaching Methods

Lectures and group discussion were used.

### 2.10 Evaluation of the Program

It was done at different levels during the implementation of the program, at the beginning of the program to know how the participants react to it, during the implementation to evaluate the teaching methods and at the end. The evaluation was done before and after completion of the program by test.

### 2.11 Ethical Consideration

Consent was obtained from the participants involved in the study after full explanation of the program.

### 2.12 Pre- and Post Tests

A structured, pretested questionnaire that composed of 15 questions which covered all aspects of the diabetes mellitus was used. After implementation of the program the post test was done to evaluate its effectiveness on the participants’ knowledge.

### 2.13 Methods of Analysis

Two tests were used: Person correlation coefficient and Spearman and Brown equations to find out the validity and reliability of the two tests. (http://geographyfieldwork.com/SpearmansRankSignificance.htm)

The equation used is:


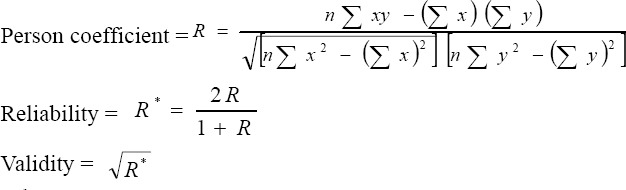


Where

n = sample size

R = Person coefficient

x = data for pretest

y = Data for posttest

The validity and reliability for diabetes health educators were 0.92 and 0.84 respectively.

### 2.14 Statistical Analysis

The data was collected by pre- and post tests and was entered using SPSS; the T-test for one group was used. P value equal or less than 0.05 was considered statistically significant.

## 3. Results

As shown in table (1) 36 diabetes health educators attended the program. There was a significant statistical difference (p=0.001) between mean of Pre and post test concerning knowledge of diabetes health educators.

## 4. Discussion

The program was designed with good contents, and the teaching methods used were well, shown by the mean and T-test results in Table 1 above. In the pre- test some questions were not answered correctly, but after the implementation of the program the participants answered the questions correctly. This indicates that the educational program had improved the health educators’ knowledge. To my knowledge there were no studies done, before, in Sudan to evaluate the knowledge of diabetes health educators after attending an educational program in order to compare the results with.

**Table 1:** The results of T-test between pretest and posttest for knowledge of diabetes health educators working at Diabetic Health Centers in Khartoum State, Sudan; 2007 – 2010 (N=36)

Group	Compare test	N	Mean	SD	Df	T	P value	Significance
Diabetes health educators	Pre test	36	44.19	7.97	35	5.25	0.001	Significant

Post test	36	52.25	4.59

## 5. Conclusion and Recommendations

The educational program improved the diabetes health educators’ knowledge. Educating and training more diabetes health educators, so that they can work in diabetic centers, hospitals and primary health care clinics, specially the rural areas; develop and equip more diabetic centers with audio tapes, video tapes, pamphlets, leaflets, magazines, and books; was recommended. The diabetes health educators should have standards, and scope of practice that organizes and standardizes the work and in-service education for diabetic health educators to update their knowledge and skills is very important.
